# Effects of a 6-Week Intermittent Hypoxia–Hyperoxia Exposure Program on Blood Pressure, Respiratory Function, Cardiac Autonomic Nervous Activity and CRP Levels in Older Adults: A Randomized Clinical Trial

**DOI:** 10.3390/sports14010042

**Published:** 2026-01-16

**Authors:** Arturo Ladriñán-Maestro, Alberto Sánchez-Sierra, María Herrera-Gómez-Platero, Jorge Sánchez-Infante

**Affiliations:** 1 Pain, Mental Health, Exercise and Technology Research Group (PAIN + MET), Faculty of Physical Therapy and Nursing, Universidad de Castilla-La Mancha, 45071 Toledo, Spain; arturo.ladrinan@uclm.es; 2 Grupo de Investigación en Fisioterapia Toledo (GIFTO), Facultad de Fisioterapia y Enfermería, Universidad de Castilla-La Mancha, 45071 Toledo, Spain; alberto.sanchez@uclm.es; 3 Grupo de Investigación en Fisioterapia Toledo (GIFTO), Instituto de Investigación Sanitaria de Castilla-La Mancha, 45071 Toledo, Spain; 4 Institute of Health and Sport Sciences, Faculty of Health Science, Universidad Francisco de Vitoria, 28223 Madrid, Spain; maria.herrera@ufv.es

**Keywords:** autonomic cardiac control, respiratory rehabilitation, respiratory muscle strength, physiotherapy, intermittent hypoxia

## Abstract

Background and Objectives: The aim of this study is to objectively evaluate the effects of a six-week intermittent hypoxic–hyperoxic exposure program on blood pressure, respiratory function, cardiac autonomic nervous activity and C Reactive Protein levels in older adults. Materials and Methods: A double-blinded randomized controlled clinical trial was conducted on twenty-two older adults. Heart rate variability, respiratory function, blood pressure, C Reactive Protein levels and oxygen saturation were measured at two time points: baseline and after 6 weeks of treatment. Results: The maximal inspiratory pressure variable increased significantly in the EG (+7.50 ± 1.72 cmH2O, *p* < 0.01, ES = 1.17), while no changes were observed in the CG. The LF/HF variable decreased significantly in the EG (−1.23 ± 0.34 n.u, *p* < 0.01, ES = 1.11), with no significant changes in the CG. The C Reactive Protein variable decreased significantly in the EG (−7.00 ± 3.07 mg/L, *p* < 0.01, ES = 1.4), with no significant changes in the CG. Conclusions: Six weeks of intermittent hypoxic–hyperoxic exposure was associated with trends toward improvements in blood pressure, respiratory function, cardiac autonomic nervous activity, and C Reactive Protein levels, compared with a placebo application of the same therapy.

## 1. Introduction

Aging is a normal and natural process that frequently leads to both functional and organic deterioration in older adults. Variables such as muscle mass, strength, functional capacity, mobility, and cognitive function may be compromised during this stage of life [1]. At the respiratory level, there is also the potential for a decline in cardiopulmonary function and increased weakness of the respiratory muscles, which can negatively impact physical fitness and quality of life [2]. Regarding inflammatory biomarkers, aging is associated with elevated serum concentrations of pro-inflammatory substances such as interleukin-6 and C-reactive protein (CRP). When these elevations persist over time, they can contribute to a state of chronic inflammation and an increased risk of developing comorbidities [3]. Given the growing aging population, the prevention and treatment of age-related diseases pose a significant challenge [4].

Oxygen consumption is closely linked to cardiorespiratory fitness and, consequently, to functionality, quality of life, and mortality risk [5]. Oxygen deprivation, known as hypoxia, has varying effects on the body. On the one hand, chronic or prolonged exposure to hypoxic stimuli can have detrimental effects, such as increased blood pressure, heart rate, systemic inflammation, and vasoconstriction. On the other hand, intermittent short-term exposure to hypoxia has been shown to induce beneficial physiological adaptations, including enhanced vasodilation, reduced oxidative stress, and improved immune function. These beneficial effects support intermittent hypoxic–hyperoxic exposure (IHHE) as a non-invasive and non-pharmacological therapeutic approach [6]. By alternating hypoxic and hyperoxic phases, IHHE allows controlled physiological stimulation while minimizing potential adverse effects associated with sustained hypoxia. In particular, the hyperoxic phase facilitates recovery from the hypoxic stimulus and may enhance antioxidant defenses and tissue oxygenation [7]. This combined exposure has been proposed as a strategy to optimize adaptive responses while maintaining safety, making IHHE a promising therapeutic alternative for diverse clinical conditions and populations, especially in individuals for whom conventional exercise-based or pharmacological interventions may be limited [8]. These contradictory findings may be explained by differences in the pattern, duration, and intensity of hypoxic exposure, as well as the characteristics of the studied populations (e.g., age, baseline cardiovascular health, or presence of comorbidities). For instance, continuous or severe hypoxia is more likely to provoke negative cardiovascular or inflammatory responses, whereas short, controlled intermittent exposures—as applied in IHHE protocols—tend to elicit adaptive responses that improve vascular and oxidative function. This nuanced understanding of the differential effects of hypoxia across experimental conditions supports the rationale for investigating IHHE as a controlled, non-invasive intervention in older adults [8]. While IHHE has been studied extensively in athletes and patients with metabolic conditions, available studies indicate that IHHE can elicit physiological responses in older individuals, such as increased erythropoietin levels [9], body composition, functional fitness, cardiovascular and bone health [10] and improve lipid profile and anti-inflammatory status [11].

Despite these potential benefits, the current evidence regarding the effects of IHHE on autonomic cardiac control, the respiratory function, or inflammatory biomarkers in the geriatric population remains limited or inconclusive. the primary aim of the present study was to evaluate the effects of a six-week IHHE program on systolic and diastolic blood pressure in older adults. Secondary outcomes included respiratory function, cardiac autonomic nervous activity, and CRP levels. Based on previous evidence, we hypothesized that IHHE will lead to reduced blood pressure, improved respiratory function, enhanced cardiac autonomic regulation, and decreased CRP levels in this population. Accordingly, our research question was: does a six-week IHHE program improve cardiovascular, respiratory, autonomic, and inflammatory outcomes in older adults?

## 2. Materials and Methods

### 2.1. Study Design

A randomized, double-blind clinical trial was conducted at the Physiotherapy Department of Residencial Montes de Toledo (Manzaneque, Spain), in accordance with the Consolidated Standards of Reporting Trials (CONSORT) guidelines. Written informed consent was obtained from all participants. A total of 22 participants were assessed for eligibility. Of these, 20 participants were included in the final analysis, following two dropouts. The study received approval from the Research Ethics Committee of the Complejo Hospitalario Universitario de Toledo (ID: 1071) and was registered at ClinicalTrials.gov (ID: NCT06686316).

### 2.2. Participants

Twenty-two older adults were recruited from specify setting, e.g., the Residencial Montes de Toledo (Manzaneque). Participants were invited to participate through informational sessions and randomly allocated to either the experimental group (EG) or the control group (CG), each receiving three sessions per week for six weeks. The EG received the IHHE treatment, whereas the CG received the sham IHHE treatment. Randomization was performed using Randomization.com software. Simple randomization was applied, without block randomization or stratification by sex or age, due to the small sample size. Both the evaluator and participants were blinded to group allocation. The inclusion criteria required participants to be over 60 years old, have no prior experience with hypoxic training, and engage in less than 150 min of physical activity per week. Exclusion criteria included any condition limiting independent walking or functionality, cognitive impairment, pulmonary hypertension, decompensated cardiac or respiratory disease, acute infection within the three months prior to enrollment or during the study period, hospitalization within the previous three months, current or past cancer diagnosis, and acute or chronic use of medications that could affect the primary or secondary outcomes, including anti-inflammatory drugs, beta-blockers, antihistamines, and antihypertensive medications. The sample size was calculated using G*Power software (version 3.1.9.2) based on systolic blood pressure values from a previous study [12] with an alpha error of 0.05, a beta error of 0.2, and a medium effect size (f = 0.25 or partial eta squared = 0.06). A 20% dropout rate was accounted for due to the study design, leading to a final required sample of 22 participants, equally distributed between the two groups (*n* = 10 per group).

### 2.3. Intervention

#### 2.3.1. Intermittent Hypoxic–Hyperoxic Exposure

The EG underwent a 6-week intervention protocol consisting of three weekly IHHE sessions (Monday, Wednesday, and Friday) using the MITOVIT^®^ Hypoxic Training System (COMMIT GmbH, Salzgitter, Germany). Each session included six cycles of 5 min of hypoxia followed by 3 min of hyperoxia. Oxygen saturation was maintained between 85 and 92% during hypoxia and above 95% during hyperoxia. FiO_2_ is automatically adjusted in real time through the device’s artificial intelligence algorithm to maintain the desired SpO_2_ range throughout the session [6]. Participants remained seated and were instructed to breathe normally throughout the exposure. Oxygen saturation and heart rate were continuously monitored at all times.

#### 2.3.2. Sham Intermittent Hypoxic–Hyperoxic Exposure

The CG followed a 6-week sham intervention protocol consisting of three weekly sham IHHE sessions (Monday, Wednesday, and Friday), with six cycles of five minutes per session, using an FiO_2_ of 21% delivered by the MITOVIT^®^ Hypoxic Training System (COMMIT GmbH, Salzgitter, Germany) [6]. Participants remained seated and were instructed to breathe normally throughout each exposure. Oxygen saturation and heart rate will be continuously monitored at all times.

### 2.4. Outcomes

The analysis of the variables was conducted at two time points: 24 h before the intervention and 24 h after the final session of IHHE or sham IHHE, in order to avoid potential acute effects induced by the intervention. The primary outcome variables were systolic and diastolic blood pressure. Secondary outcomes included heart rate variability indices (SDNN, RMSSD, LF/HF), respiratory function parameters (FVC, FEV_1_, MIP), and C-reactive protein levels.

#### 2.4.1. Heart Rate Variability

The analysis of HRV was conducted using a heart rate monitor (Polar H10; Polar Electro Oy, Kempele, Finland). Cardiac electrical signals were monitored with a band placed on the chest for 5 min with the subject in supine position. Subjects are instructed not to speak or make voluntary movements during this analysis [13]. The data were processed using Kubios HRV Analysis Software version 3.1.0 for Windows (Biomedical Signal and Medical Imaging Analysis Group, Department of Applied Physics, University of Kuopio, Kuopio Finland). Five parameters were extracted: Heart rate (HR), RR Interval measured in milliseconds (R-Ri), the standard deviation of all normal-to-normal intervals (SDNN) measured in milliseconds, the sympathovagal balance index calculated as the LF/HF ratio, and the root mean square of successive differences between adjacent normal-to-normal intervals measured in milliseconds (RMSSD). HR, SDNN, and RR interval provide measures of overall autonomic activity, RMSSD reflects parasympathetic (vagal) modulation, and the LF/HF ratio indicates sympathovagal balance. It should be noted that this method does not allow for direct estimation of cardiac sympathetic activity

#### 2.4.2. Arterial Blood Pressure

Both systolic blood pressure (SBP) and diastolic blood pressure (DBP) were assessed using a standard digital sphygmomanometer (Omron HEM-705CP, Omron Healthcare, Inc., Lake Forest, IL, USA). The subjects rested for 10 min prior to the measurements to stabilize their blood pressure. Two measurements were taken, and the average values were selected [14].

#### 2.4.3. Respiratory Muscle Strength

Maximal inspiratory pressure (MIP) was measured using the MicroRPM^®^ Respiratory Pressure Measurement Device (MicroMedical, Kent, UK) while participants were seated. To prevent nasal airflow, a nose clip was applied. Participants rested for one minute between attempts and performed up to six maneuvers. The highest reproducible value from three trials, with a variability of less than 5%, was recorded [15].

#### 2.4.4. Pulmonary Function

Pulmonary function assessments were performed using the Air Smart Spirometer (Pond Healthcare Innovation, Stockholm Sweden) in accordance with the guidelines established by the American Thoracic Society (ATS) and the European Respiratory Society (ERS). The parameters measured included Forced Vital Capacity (FVC) and Forced Expiratory Volume in one second (FEV1), along with the FEV1/FVC ratio [16].

#### 2.4.5. C Reactive Protein

Standard CRP concentrations were measured using a standard immunoassay method. A nurse collected venous blood samples from the antecubital vein after a minimum of 10 h of overnight fasting. The samples were centrifuged at 1800 rpm to extract the serum, which was then stored at −80 °C until analysis. CRP concentrations were determined using an ELISA microplate reader (SpectraMax PLUS 384, Molecular Devices, San Jose, CA, USA), following the manufacturer’s instructions [17].

#### 2.4.6. Arterial Oxygen Saturation

Arterial oxygen saturation (SpO_2_) was assessed using a Nonin^®^ 3230 pulse oximeter (Nonin Medical, Inc., Plymouth, MN, USA). Measurements were performed over a 10 min period with participants seated, and the average values were recorded [18].

### 2.5. Statistical Analysis

Statistical analysis was performed using IBM SPSS Statistics version 22.0, with a significance threshold set at *p* < 0.05. The normality of each variable was assessed using the Shapiro–Wilk test, confirming that all variables followed a normal distribution. Descriptive statistics were utilized to summarize the demographic data, with results expressed as mean ± standard deviation (SD). To analyze the outcome variables, a two-way repeated measures ANOVA was conducted to assess the interaction between the Experimental and Control groups at the baseline and 6-week follow-up time points. Significant differences were further examined through post hoc analyses using Bonferroni-adjusted multiple comparisons. Effect sizes (ES) were determined using Cohen’s scale [19]: small (<0.20), medium (0.50), and large (>0.80).

## 3. Results

### 3.1. Demographic Data

**Figure 1:**
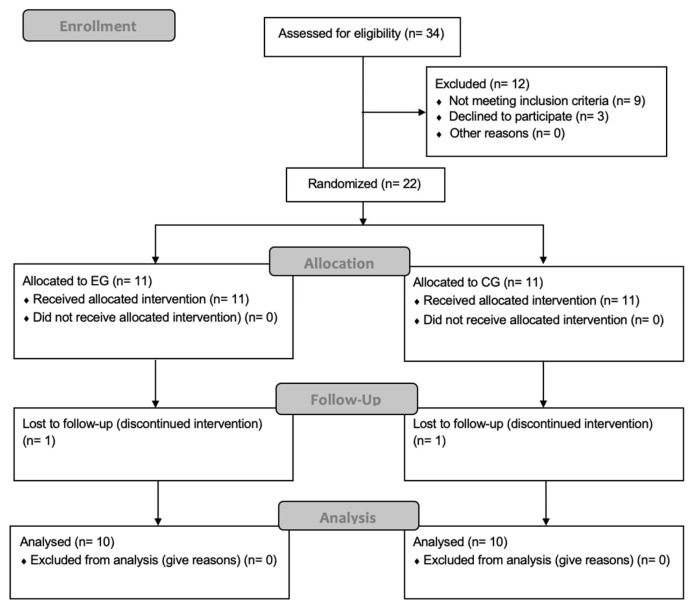
Study flow chart.

In December 2024, twenty-two participants were recruited, and twenty completed the study and were included in the final analysis, with their participation taking place between December 2024 and January 2025. Participants were assigned to the EG (7 men, 4 women) and the CG (7 men, 4 women). Two participants withdrew due to reasons related to the intervention and measurements. No adverse events or safety issues were observed during any of the sessions. A CONSORT flow chart is provided (Figure 1). No significant differences in demographic characteristics were observed between the EG and CG (Table 1).

### 3.2. Changes in Outcomes

**Figure 2:**
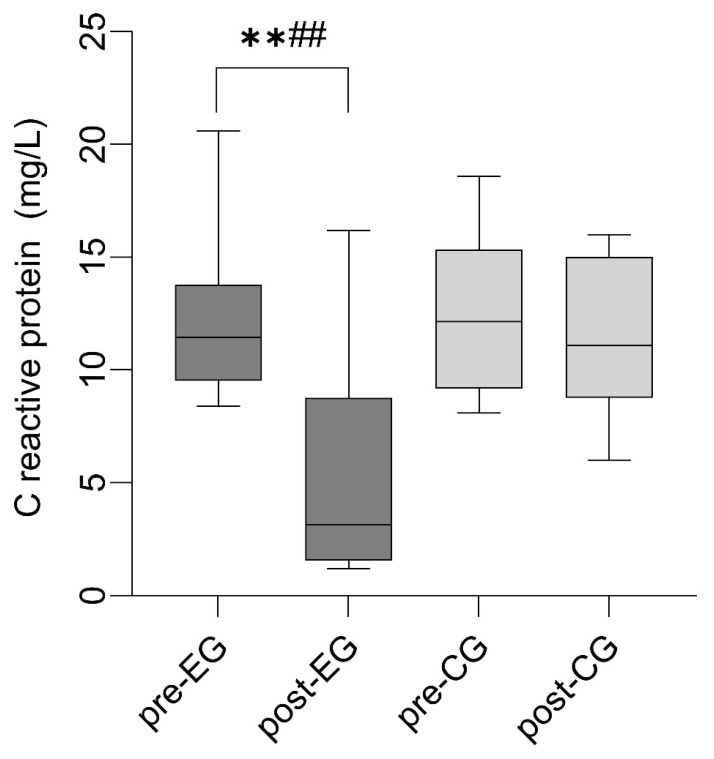
Study Box-and-whisker plot illustrating C-reactive protein levels in the experimental and control groups at baseline and after a 6-week intervention. The box represents the interquartile range (Q1–Q3), the central line denotes the median, and the whiskers extend to the minimum and maximum values. ** *p* < 0.01, 6 weeks follow-up, with baseline. ## *p* < 0.01, comparisons between the EG and CG at corresponding time points.

Results for outcomes are presented in Table 2 and Table 3 and Figure 2.

In the analysis of MIP variable, the EG showed a significant increase from baseline to post-treatment of +7.50 ± 1.72 cmH2O (*p* < 0.01, ES = 1.17; 95% CI for the difference = 5.65 to 8.35), indicating a positive and clinically relevant effect of the intervention. In contrast, no significant changes were observed in the CG, suggesting that the observed improvement was specifically associated with the IHHE intervention. The large effect size further supports the practical relevance of this finding.

With respect to systemic inflammation, CRP levels in the EG decreased significantly from baseline to post-treatment of −7.00 ± 3.07 mg/L (*p* < 0.01, ES = 1.40; 95% CI for the difference = −8.21 to −3.79), whereas no significant changes were observed in the CG. This pattern indicates a consistent reduction in CRP levels associated with the intervention.

Similarly, the LF/HF ratio, reflecting cardiac autonomic balance, showed a significant reduction in the EG following the intervention of −1.23 ± 0.34% (*p* < 0.01, ES = 1.11; 95% CI for the difference = −1.49 to −0.92), with no significant changes observed in the CG. This finding suggests a shift toward a more favorable autonomic profile after the IHHE program.

## 4. Discussion

The present findings directly address our research question of whether a six-week IHHE program can improve cardiovascular, respiratory, autonomic, and inflammatory outcomes in older adults. The observed reductions in blood pressure and LF/HF ratio, together with the increase in maximal inspiratory pressure, support our hypothesis that IHHE may induce favorable cardiovascular, autonomic, and respiratory adaptations in this population. However, the absence of significant changes in some respiratory parameters and the exploratory nature of the CRP findings indicate that these effects are not uniform and should be interpreted with caution. The results of this study suggest that six weeks of IHHE improve HRV, blood pressure, respiratory function, and systemic inflammation compared to a placebo application of the same intervention.

It is well-documented that, even in older adults, high-intensity exercise may induce transient reductions in arterial oxygen saturation, with SpO_2_ values occasionally falling below 90% in well-conditioned individuals, without evidence of underlying pathology or clinically relevant adverse effects. This physiological response reflects the increased metabolic and ventilatory demands imposed by strenuous exercise and does not preclude the well-established health benefits of physical training in this population [20]. In this context, intermittent hypoxic exposure induces controlled and transient reductions in oxygen availability that are comparable in magnitude to those observed during high-intensity exercise, while being applied under standardized and closely monitored conditions. Therefore, when appropriately prescribed and supervised, intermittent hypoxic exposure appears to represent a safe and well-tolerated stimulus capable of promoting physiological adaptations in older adults.

Regarding respiratory function, inspiratory muscle strength was assessed using MIP, while pulmonary function was evaluated through spirometry. Our results suggest improvements in MIP, FVC, and FEV1/FVC, which may translate into enhanced inspiratory muscle strength and vital capacity. These effects could be attributed to increased mitochondrial biogenesis and efficiency in respiratory muscles [7], as well as enhanced respiratory plasticity of the phrenic, vagus, and intercostal nerves, mediated by the serotonergic system [21], leading to improved synaptic transmission to respiratory motor neurons [22]. Hypoxia-Inducible Factor-1 alpha (HIF-1α) is a key transcription factor mediating cellular adaptation to reduced oxygen availability. Under hypoxic conditions, HIF-1α stabilizes and translocates to the nucleus to activate hypoxia-responsive elements, which regulate genes involved in metabolic reprogramming, angiogenesis, and mitochondrial function. HIF-1α has been shown to influence mitochondrial respiration, structure, and biogenesis by modulating the expression of genes that affect mitochondrial metabolism and respiratory chain efficiency, thereby contributing to adaptive changes in muscle metabolism under hypoxia. These processes may contribute to enhanced ventilatory efficiency and respiratory muscle function following intermittent hypoxic–hyperoxic exposure, although further research is needed to directly confirm these pathways in older adults [23]. MIP is a critical variable due to its strong association with HRV [24], maximal oxygen consumption and lactate levels [25,26], while spirometry parameters appear to correlate with functionality and exercise tolerance [27,28]. From a clinical perspective, an increase of approximately 7 cmH2O in MIP may represent a meaningful improvement in inspiratory muscle strength, which has been associated with reduced dyspnea, improved functional capacity, and greater tolerance to daily physical activities in older adults. These mechanisms remain hypothetical and speculative, and further research is needed to confirm their relevance in older adults undergoing IHHE.

Regarding HRV outcomes, our results suggest a generalized improvement in all variables was observed, with a reduction in sympathetic activity and an increase in vagal tone, consistent with previous studies [29]. This effect could be attributed to the parasympathetic activation induced by hypoxia [30], possibly via greater activation of the nucleus tractus solitarius [31], which is a key brainstem integrative center that receives afferent input from cardiovascular and respiratory receptors and coordinates autonomic reflexes to regulate blood pressure, heart rate, and breathing. This central role in cardiorespiratory integration is well-established in both human and animal studies [32]. These mechanisms may also explain the observed improvements in blood pressure. Our findings align with those of previous studies [33,34,35], suggesting that these benefits may be driven by enhanced HRV, increased angiogenesis, and vasodilation, mediated by the release of hypoxia-inducible factor-1 alpha [36] and greater nitric oxide availability, which reduces vascular resistance and consequently lowers blood pressure [37]. As for SpO_2_, the observed improvements might be associated with a reduction in oxidative stress and increased nitric oxide bioavailability, potentially reflecting enhanced endothelial function, reduced endothelial reactivity, and improved gas exchange [38].

CRP was selected as the primary marker of systemic inflammation due to its clinical relevance, widespread use, and well-standardized measurement in both research and clinical settings. While other inflammatory cytokines such as IL-6 or TNF-α provide additional mechanistic insights, they often exhibit higher biological variability and require more complex assays, which may limit their feasibility in older adult populations. CRP has been used in previous studies involving intermittent hypoxia in older adults, where significant changes in CRP concentrations were observed in response to hypoxic conditioning protocols [39] and following long-term intermittent hypoxia exposure [17]. Our results suggest a decrease in serum CRP levels, which may indicate a potential reduction in systemic inflammation, consistent with previous research [11]. Clinically, a reduction of approximately 7 mg/L in CRP may reflect a relevant decrease in low-grade systemic inflammation, which has been associated with lower cardiovascular and cardiometabolic risk in older populations, although this interpretation should be made cautiously given the absence of long-term follow-up. These effects could be explained by a modulation of inflammatory pathways rather than a direct anti-inflammatory effect per se. In this context, hypoxia-related interventions have been suggested to influence the balance between pro- and anti-inflammatory mediators, potentially through mechanisms involving hypoxia-inducible factors, oxidative stress regulation, and autonomic nervous system adaptations. Specifically, a suppression of pro-inflammatory cytokines such as tumor necrosis factor-alpha and interleukin-4 has been reported [40], together with an increased release of anti-inflammatory mediators, including interleukin-10 and adenosine [41]. Such mechanisms could contribute to a reduction in low-grade systemic inflammation, which may be reflected by changes in circulating CRP levels. However, given the limited evidence in sedentary older adults and the exploratory nature of CRP assessment in the present study, these findings should be interpreted with caution and considered hypothesis-generating rather than confirmatory.

This study has several limitations that should be acknowledged. Although the sample size was appropriate, future studies with larger sample sizes and diverse clinical populations are needed to confirm these findings. The sample included only sedentary older adults and was recruited from a single center, which may limit the generalizability of the findings. In addition, the study population lacked clinical diversity, as participants were generally healthy and free of significant comorbidities. Furthermore, C-reactive protein is a non-specific marker of systemic inflammation, and the absence of parallel cytokine measurements (e.g., IL-6, TNF-α) limits mechanistic interpretation of the observed inflammatory changes. Future research should investigate the effects of intermittent hypoxic–hyperoxic exposure in more diverse populations, including physically active older adults and individuals with common age-related comorbidities, to determine the broader applicability and potential therapeutic benefits of this intervention. Additionally, only one pre-treatment measurement and one post-treatment measurement (after six weeks) were performed; incorporating multiple assessments throughout the intervention and follow-up periods could have provided a more detailed understanding of the therapy’s effects over time. Future studies could include an assessment of participants’ perception of the intervention, such as a post-intervention questionnaire, to further evaluate the effectiveness of blinding and ensure that the sham protocol is indistinguishable from the active intervention. From a clinical applicability perspective, the findings suggest promising benefits, as IHHE is a passive and well-tolerated intervention that does not impose mechanical stress while offering cardiovascular, autonomic, and metabolic advantages.

## 5. Conclusions

In conclusion, six weeks of IHHE was associated with trends toward improvements in blood pressure, respiratory function, cardiac autonomic nervous activity, and CRP levels, compared with a placebo application of the same therapy.

## Figures and Tables

**Table 1:** Demographic characteristics of subject.

	EG (*n* = 10)	CG (*n* = 10)	*p*
Sex (male/female)	7/3	6/4	
Age (yrs)	71.20 ± 6.11	71.70 ± 5.27	n.s
Weight (kg)	70.40 ± 6.98	69.30 ± 6.77	n.s
Height (cm)	171.40 ± 5.78	169.00 ± 7.16	n.s

EG, Experimental Group; CG, Control group.

**Table 2:** Outcome measurements of respiratory variables.

	Baseline	6 Weeks Follow-Up		f	*p*	n^2^	Pot
MIP (cmH2O)							
EG	66.20 ± 4.76	73.70 ± 5.58 **	Group	1.47	0.24	0.08	0.21
CG	66.60 ± 6.40	67.10 ± 6.19 #	Time	155.68	<0.01	0.90	1
			Group × Time	119.19	<0.01	0.90	1
FVC (%)							
EG	76.13 ± 12.24	85.97 ± 8.67 **	Group	3.16	0.09	0.15	0.39
CG	73.68 ± 7.78	73.95 ± 8.25 ##	Time	22.95	<0.01	0.56	1
			Group × Time	20.57	<0.01	0.53	0.99
FEV1 (%)							
EG	70.03 ± 15.55	70.03 ± 16.33	Group	<0.01	0.97	<0.01	0.05
CG	70.46 ± 13.27	69.08 ± 12.16	Time	0.97	0.34	0.05	0.15
			Group × Time	0.95	0.34	0.05	0.15
FEV1/FVC (%)							
EG	88.31 ± 8.66	77.55 ± 5.73 **	Group	2.74	0.12	0.13	0.35
CG	91.01 ± 15.22	91.35 ± 14.34 #	Time	10.95	<0.01	0.38	0.88
			Group × Time	12.45	<0.01	0.41	0.92

EG, Experimental group; CG, Control group; MIP, Maximal inspiratory pressure; FVC, Forced Vital Capacity; FEV1, Forced Expiratory Volume in 1 s; FEV1/FVC, Tiffeneau-Pinelli Index. Values are mean ± SD. ** *p* < 0.01, 6 weeks follow-up, with baseline # *p* < 0.05, ## *p* < 0.01, comparisons between the EG and CG at corresponding time points.

**Table 3:** Outcome measurements of heart rate variability.

	Baseline	6 Weeks Follow-Up		f	*p*	n^2^	Pot
RR (ms)							
EG	794.12 ± 43.65	952.24 ± 44.31 **	Group	0.23	0.64	0.01	0.07
CG	852.63 ± 123.98	854.68 ± 124.09 #	Time	136.46	<0.01	0.88	1
			Group × Time	129.57	<0.01	0.88	1
SDNN (ms)							
EG	47.52 ± 25.61	59.58 ± 27.78 **	Group	0.04	0.85	<0.01	0.05
CG	52.17 ± 15.93	51.16 ± 16.87	Time	35.97	<0.01	0.67	1
			Group × Time	50.37	<0.01	0.74	1
LF/HF (n.u)							
EG	1.99 ± 0.67	0.76 ± 0.43 **	Group	0.79	0.39	0.04	0.14
CG	1.70 ± 1.01	1.68 ± 0.94 #	Time	86.46	<0.01	0.83	1
			Group × Time	79.95	<0.01	0.82	1
RMSSD (ms)							
EG	27.92 ± 14.27	51.73 ± 11.41 **	Group	0.21	0.65	0.01	0.07
CG	37.71 ± 9.09	37.64 ± 9.16 ##	Time	46.59	<0.01	0.72	1
			Group × Time	47.09	<0.01	0.72	1
SpO_2_ (%)							
EG	93.20 ± 1.62	96.10 ± 1.73 **	Group	0.53	0.48	0.03	0.11
CG	94.20 ± 2.57	93.90 ± 2.13 #	Time	11.10	<0.01	0.38	0.88
			Group × Time	16.76	<0.01	0.48	0.97
HR (bpm)							
EG	74.20 ± 4.44	64.20 ± 3.97 **	Group	<0.01	0.95	<0.01	0.05
CG	69.40 ± 6.72	69.30 ± 6.96	Time	79.90	<0.01	0.82	1
			Group × Time	76.77	<0.01	0.81	1
SBP (mmHg)							
EG	135.20 ± 11.34	108.40 ± 9.18 **	Group	0.30	0.59	0.30	0.08
CG	125.30 ± 11.74	123.50 ± 11.66 ##	Time	128.88	<0.01	0.88	1
			Group × Time	98.48	<0.01	0.85	1
DBP (mmHg)							
EG	76.80 ± 5.59	65.90 ± 3.31 **	Group	0.03	0.87	<0.01	0.05
CG	71.60 ± 8.38	70.20 ± 6.63	Time	37.10	<0.01	0.67	1
			Group × Time	22.11	<0.01	0.55	0.99

EG, Experimental group; CG, Control group; RR, Interval (R-Ri); SDNN, standard deviation of all normal-to-normal intervals; LF/HF, the sympathovagal balance index as the ratio between low and high frequency power; RMSSD, the square root of the mean of the sum of squared differences between adjacent normal-to-normal intervals; SpO_2_, arterial oxygen saturation; HR, heart rate; SBP, systolic blood pressure; DBP, diastolic blood pressure. Values are mean ± SD. ** *p* < 0.01, 6 weeks follow-up, with baseline # *p* < 0.05, ## *p* < 0.01, comparisons between the EG and CG at corresponding time points.

## Data Availability

The data presented in this study are available on request from the corresponding author.

## Abbreviations

The following abbreviations are used in this manuscript:

EG	Experimental Group
CG	Control Group
IHHE	Intermittent Hypoxic–Hyperoxic Exposure
HRV	Heart Rate Variability
MIP	Maximal Inspiratory Pressure
FVC	Forced Vital capacity
FEV1	Forced Expiratory Volume in One Second
CRP	C-Reactive Protein
SBP	Systolic Blood Pressure
DBP	Diastolic Blood Pressure
SpO2	Arterial Oxygen Saturation
